# Risk factors for severe Covid-19 breakthrough infections: an observational longitudinal study

**DOI:** 10.1186/s12879-022-07859-5

**Published:** 2022-11-28

**Authors:** Sihem Ben Fredj, Rim Ghammem, Nawel Zammit, Amani Maatouk, Nihel Haddad, Nouha Haddad, Manel Kachroudi, Senda Rebai, Hafsia Laadhari, Mohamed Mizouni Ghodhbani, Jihen Maatoug, Hassen Ghannem

**Affiliations:** 1 Faculty of Medicine of Sousse, University of Sousse, 4000 Sousse, Tunisia; 2 Department of Epidemiology “LR19SP03”, Farhat Hached University Hospital, 4000 Sousse, Tunisia; 3 Ministry of Health, Regional Health Office of Sousse, Sousse, Tunisia

**Keywords:** COVID-19, COVID-19 breakthrough infections, COVID-19 vaccines, Risk factors

## Abstract

**Background:**

The drive to vaccinate large populations is nowadays the main instrument for combating the pandemic and preventing serious disease and death. However, breakthrough infection (post-vaccination infection) still happens after vaccination among fully vaccinated people. We aimed to assess the severity outcomes and to determine its associated factors among vaccinated COVID-19 cases in the governorate of Sousse, Tunisia.

**Methods:**

We carried out a five-month observational longitudinal study including all the population of Sousse. Confirmed infections of SARS-CoV-2 and the vaccination status are recorded in the daily COVID- 19 database of the Regional Office of the Tunisian Ministry of Health. We included all post-vaccination COVID-19 cases for the analysis of the COVID-19 serious outcomes. Data were collected via 15-min telephonic call interviews conducted by trained interviewers. Descriptive analysis with calculating incidence rates of confirmed COVID-19 cases per 100,000 inhabitants was conducted. In binary logistic regression, adjusted odds ratios along with 95% intervals confidence were performed to determine factors related to severe or critical COVID-19.

**Results:**

As of 31 July 2021, 107,545 persons over 19 years old have received at least one dose of COVID-19 vaccination. Among the vaccinated population, we traced and included 765 breakthrough infection cases, and the incidence rate was 711.3 per week. The majority were female (sex-ratio = 0.8), and the average age of the overall cases was 55.7 years. The prevalence of severe or critical cases in vaccinated COVID-19 patients occurs in 10.8% of cases. Patients with a medical history of cardiovascular diseases had more than two times increased odds to have a severe or critical disease. We also found the highest self-estimation of adherence to preventive measures was inversely correlated to serious cases and having an incomplete vaccination schema was strongly associated with complications.

**Conclusions:**

We tried to provide evidence about the breakthrough infections to improve measures of prevention and control of COVID-19. Boosting immunity for vulnerable patients added to maintaining and promoting preventive measures are not only essential to prevent severe cases of breakthrough infections of COVID-19, but also other influenza-like diseases.

## Background

As of August 31st, 2021, the COVID-19(Coronavirus disease 2019) has affected more than 214 million people and resulted in 3.5 million deaths across almost all countries [1]. In most cases, the symptoms of COVID-19 are mild. However, in certain cases, the disease develops into severe pneumonia and multiple organ failure with a mortality rate of 3.7% [4].The implementation of preventive measures such as infection control practices, social distancing, and border restrictions did not provide a feasible long-term solution [2].Therefore, the emergency use of authorized vaccines was mandatory. The drive to vaccinate large populations or on other terms create a non-natural “herd immunity” is nowadays the main instrument for combating the pandemic and preventing serious disease and death [3].The first vaccination campaigns began in mid-December 2020 in Europe, the Middle East, and North America [4].There are, currently, four main categories of vaccines utilizing a range of established and new vaccine technologies: whole in activated virus (e.g. Sinopharm, Bharat Biotech), Protein Subunit (e.g. Novavax, Sanofi Pasteur), Viral Vector (e.g. Sputnik V, Janssen, AstraZeneca) and RNA based vaccine (e.g. Pfizer/BioNTech, Moderna) [5].Vaccination programs have known successful progress amongst the world despite scientific debate of the variable efficacy reported with different vaccines [6–11].However, breakthrough infection (post-vaccination infection)still happens after vaccination among fully vaccinated people with changing patterns according to the predominant virus of concern (VOC) [7, 12].In addition, several studies found that not fully vaccinated persons were more likely tobe affected by critical COVID-19 illness leading to hospitalizations and deaths compared with fully vaccinated people [13–15].Tunisia knew its first epidemic phase of COVID-19 in March 2020 making it mandatory to implement special emergency measures, which help slow down the epidemic spread. However, this epidemic has known four waves resulted in a substantial rise in deaths in the country. As of December 1st, 2021, Tunisia had more than 718,000 reported cases, which resulted in25000 deaths [16].The Tunisian government's COVID-19 citizen vaccination program has begun in early March 2021, and preferentially targets healthcare professionals, individuals aged over 60 years old, individuals with a disease that increases the risk of complication and death, etc. [17]. The vaccination campaign achieved46.6% (21.9–61.5%) fully vaccinated coverage of the population over 15 years on August 31st, 2021 [18].As the vaccination program is gradually expanding its target population, the concerns regarding the ability of the vaccines to decrease the serious cases are increasing.In this paper, we aimed to assess the severity outcomes and to determine its associated factors among vaccinated COVID-19 cases between March and August 2021 in the governorate of Sousse, Tunisia.

## Methods

### Setting

The governorate of Sousse is located in the Middle Eastern of Tunisia with a population size of 737027inhabitantsrepresenting nearly5.76% of the Tunisian population [19].The first confirmed case was reported by March 16th, 2020, and a total of 42 033was reported by July 31st, 2021.Since the early stage of the COVID-19 outbreak, an intensified surveillance was implemented across the governorate of Sousse to detect suspected and confirmed COVID-19 cases, and their close contacts following standardized protocols released by the National Health Commission of Tunisia. Suspected and confirmed COVID-19 cases were defined based on the Diagnosis and Treatment scheme of COVID-19, and close contacts were defined by the Prevention and Control Scheme of COVID-19. These two schemes were released by the National Health Commission of Tunisia.

### Study design and sampling

All confirmed infections of SARS-CoV-2 (severe acute respiratory syndrome coronavirus 2**)** are recorded in the daily COVID- 19 confirmed cases database of the Regional Office of the Tunisian Ministry of Health that provides daily updates of cases number in the governorate of Sousse. This database contains individual-level data on infected patients as the following variables: age, gender, date of reporting case, date of symptom onset, and vaccination status. We conducted a five-month observational longitudinal study starting on March 1st, 2021. It concerned all patients with confirmed SARS-CoV-2 infection and having been vaccinated before the infection episode. We included all COVID-19 patients who had already been vaccinated, and we called them for data collection after their agreement.

A citizen who wishes to be vaccinated registers on the national vaccination platform. Then, he receives a message on his phone to inform him of the date, place and type of vaccine according to its availability and international protocols. The definition of complete vaccination depends on the vaccine type: mRNA vaccine or an inactivated vaccine.

Over the study period, the vaccination strategy targeted the population aged more than 18 years old. Vaccinating younger age groups was not an initial plan. The vaccination of Children over 12 years old started in October 2021. Therefore, we included only adults.

Subjects who had been infected before the vaccination antiCOVID-19 were excluded.

### Study outcome and definition

A confirmed case of COVID-19 is defined as an individual, symptomatic, or not, with a respiratory sample positive for SARS-Cov-19 using an antigenic rapid test and/or laboratory-based real-time Reverse Transcriptase Polymerase Chain Reaction (RT-PCR) essays in one nasopharyngeal sample [20]. If the antigenic test was negative, a positive RT-PCR was required to confirm COVID-19 infection. All cases were confirmed by PCR. COVID-19 disease is considered severe if the disease required oxygen therapy. Critical COVID-19 disease is an infection leading to ICU (intensive care unit) hospitalization or to death.

The Regional Office of the Tunisian Ministry of Health has been aligned with the national strategy of COVID-19 surveillance. The regional office implemented a prevention campaign in which the population was motivated to go testing if they had symptoms and healthcare professionals to be vigilant and perform the COVID-19 testing. Besides, two screening types were used: active and passive screening. Active screening for detection covid-19 in the border points, contact tracing, and clusters. Passive screening for detection covid-19 in primary health care centers, hospital departments, private medical offices, emergencies, pharmacies, and private Laboratories. This regional strategy ensured the detection of most COVID-19 cases in the governorate of Sousse. The escaped COVID-19 cases would be a small proportion.

The infection confirmation passes through a well-codified system. the authorized institutions or offices declare the daily positive tests to the Regional Office of the Tunisian Ministry of Health. A trained agent verifies the list of COVID-19 cases by calling the patients and asking them for information. Then the patient is validated and recorded in the regional database.

Definitely, there were several cases of reinfection. A patient would be recorded multiple times if he was reinfected. However, during the period of our study, we did not find a reinfection case in the study population.

Breakthrough infection was defined as a Covid-19 infection occurring ≥ 14 days after a full dose of Covid-19 vaccine.

### Data collection

Data were collected via 15-min telephonic call interviews conducted by trained interviewers at the department of Epidemiology of Sousse. Questions gathered information about sociodemographic (age, gender, district, occupation), clinical characteristics (medical history, tobacco use, Self-estimation of adherence to preventive measure, symptoms), and probable complication (oxygen use, hospitalization, and death). Each household member was called individually, whether he was hospitalized or not. Nevertheless, a household member answered the survey for the household who was dead or hospitalized in the ICU.

### Statistical analysis

Data wereanalyzed using the Statistical Package for the Social Sciences (IBM SPSS 22.0). For qualitative variables, we calculated simple frequencies and relative frequencies (percentages). For the quantitative variables, we calculated means and standard deviations, and we determined the extreme values (minimum and maximum).Percentage comparisons were made by the Chi-square test. Odds ratios (ORs) and their 95% confidence intervals (CIs) were calculated. Standard univariate/bivariate comparisons of continuous measures (student test) were used to compare means. Attack rate was calculated as the percentage of confirmed cases to be infectedwithSARS-COV-2 and expressed as cases per 100 000 inhabitants. We estimated the specific incidence rates (SIR) of confirmed cases by vaccine type. Logistic regression was also conducted to estimate the risk factors forsevere or critical cases of COVID-19.

## Results

### Epidemiologic characteristics of COVID-19 in Sousse

**Fig. 1:**
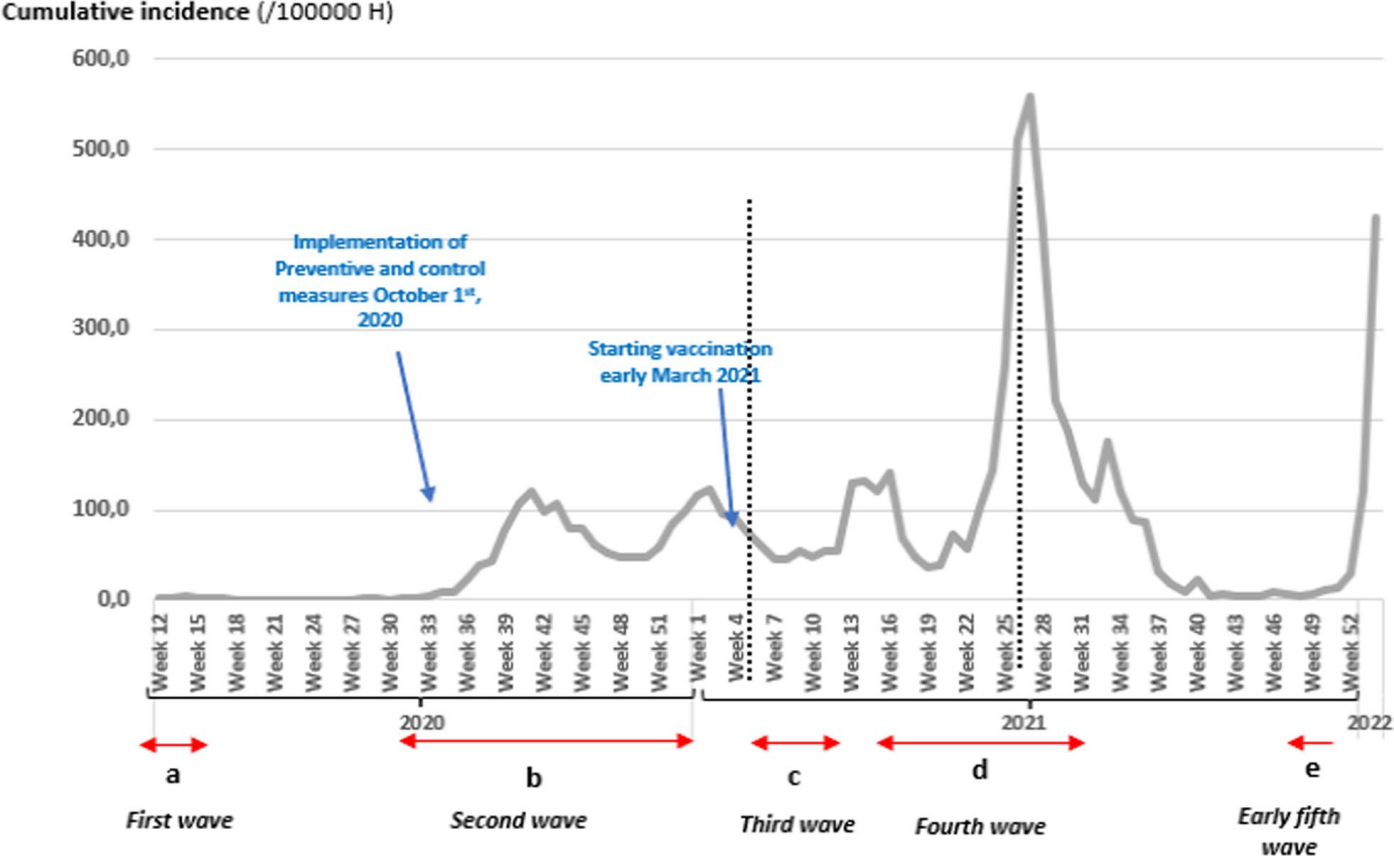
Weekly cumulative incidence curve of COVID-19 cases since March 2020 in Sousse, Tunisia, 2021

Over the sixteen months since the first case, out of the 154,729 tests performed 41,766 COVID-19 cases were confirmed from March 16th, 2020 to July 31st, 2021 in Sousse with a global cumulative incidence of 5631.2. This trend has been increased obviously since week 34/2020 and peaked in Week 27/2021 (early July); incidence = 557.6. Over the study period, the observed cumulative incidence for the cases of COVID19 was 3484 (Fig. 1).

**Fig. 2:**
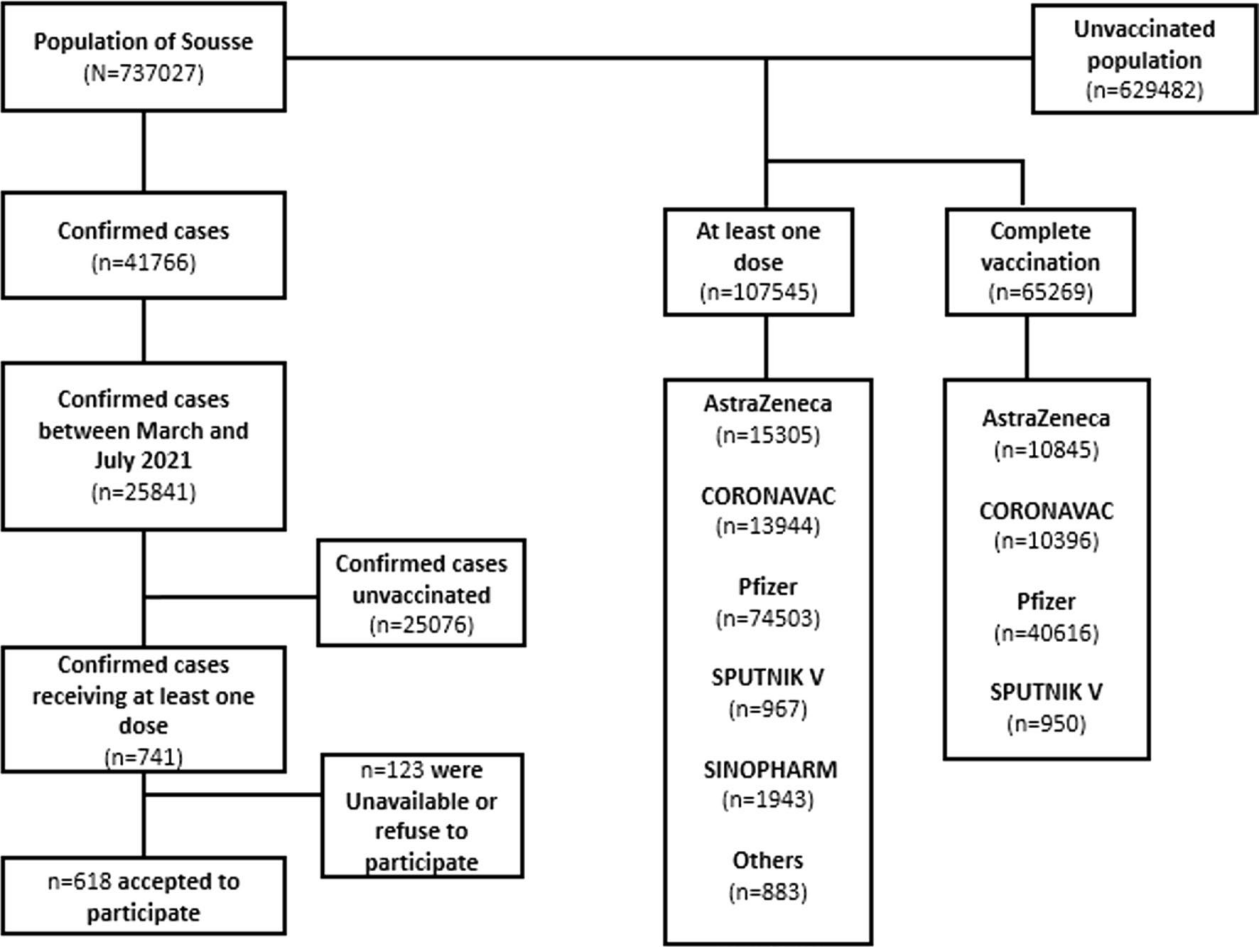
Flowchart of the inclusion process of the study population, Sousse, Tunisia, 2021

As demonstrated in Fig. 2 as of 31 July 2021, 107,545 persons over 19 years old have received at least one dose of COVID-19 vaccination. The majority received Pfizer (n = 74,503), followed by AstraZeneca (n = 15,305), CORONAVAC (n = 13,944), SINOPHARM (n = 1943) and SPUTNIK V (n = 967) and other vaccines (n = 883). Among the vaccinated population, we traced and included 765 breakthrough infection cases and the incidence rate was 711.3.

### Characteristics of patients with breakthrough COVID-19 infection

The 765 breakthrough infection cases were contacted, 123 were excluded, due to missing calls or refusing to participate, leaving 618 (80.7%) for the analysis Table 1 details their baseline characteristics. The majority were female (sex-ratio = 0.8), and the average age of the overall cases was 55.7 years(standard deviation: 14.5; range:19–91).

**Table 1:** Socio-demographic characteristics of the study population, Sousse, Tunisia, 2021

	N	%
Age (years)		
≤ 40	95	15.4
40–49	70	11.3
50–59	198	32.0
60–74	206	33.4
≥ 75	49	7.9
Gender		
Female	347	56.4
Male	271	43.6
District		
Sousse-Medina	73	11.8
Sousse-Riadh	86	13.9
Sousse-Jawhara	116	18.6
Sidi Abdelhamid	14	2.3
Hammam Sousse	60	09.9
Akouda	35	5.8
KalaâKebira	48	5.8
Sid Bou Ali	24	4.0
Hergla	9	1.5
Enfidha	17	2.8
Bouficha	10	1.6
Kondar	2	0.3
Sidi El Hèni	8	1.3
Msaken	68	11.0
KalaâSeghira	44	7.0
Zaouia-Ksiba-Thrayet	4	0.6
Occupation		
Healthcare workers	122	19.6
Other	496	80.4

Nearly half (399;49%) of participants had comorbidities, 19.6% were healthcare workers and 17.9% were smokers.On one hand,the majority (70%; n = 424)of cases received at least one dose of Pfizer followed by CORONAVAC (15.6%; n = 96). On the other hand, we found a higher incidence rate of COVID-19 among those who had been vaccinated with SPUTNIK V (SIR = 1551.2) followed by SINOPHARM (SIR = 823.7)58 patients reported a poor adherence to preventive measures whereas 38.6% reported high respect to the preventive measures. COVID-19 led to hospitalization in 8.1% of cases, hospitalization in intensive care units in 2.1% of cases, and death in 1.8% of cases. The clinical assessment revealed that only 8.2% (51 of 642) were severe (Table 2).

**Table 2:** Clinical characteristics of the study population, Sousse, Tunisia, 2021

	N	%	Median (IQ25%-IQ75%)	Specific cumulative incidence rate/100000 inhabitants
Comorbidities				
No	317	51.0		
Cardiovascular diseases	220	35.4		
Diabetes	139	22.4		
Chronic respiratory diseases	25	4.0		
Immunity system diseases	15	2.4		
Others*	40	6.5		
Tobacco use				
Yes	111	17.9		
No	510	82.1		
Vaccination schema				
Incomplete	449	72.3		1062.1
Complete	172	27.7		263.5
Vaccine				
AstraZeneca	57	9.3		379.0
CoronaVac	98	15.6		702.4
Pfizer BioNTech	424	68.9		581.2
Sinopharm	16	2.7		823.7
Sputnik	15	2.4		1551.2
Others (Moderna and Janssen)	7	1.1		792.7
Categories of vaccines**				
RNA based vaccine	425	69.3		
Viral Vector	74	12.1		
Inactivated virus	114	18.6		
Self-estimation of adherence to preventive measure				
Poor (score ≤ 4)	58	9.3		
Medium (5 ≤ score ≤ 7)	323	52.0		
High (score ≥ 8)	240	38.6		
Delay first dose and COVID19 infection			19 (10–31)	
Delay second dose and COVID19 infection			53 (19–73)	
Symptoms				
Yes	539	86.8		
No	82	13.2		
Severity of disease				
Asymptomatic or mild disease	562	91.0		
Home care oxygen or non- ICU*** hospitalization	43	6.9		
ICU*** hospitalization	13	2.1		
Death	11	1.8		

*Cancer, dysthyroid, chronic renal disease

**RNA based vaccine: Pfizer BioNtech and Moderna; Viral Vector: Sputnik, AstraZeneca, and Janssen; inactivated virus: Sinopharm

***ICU Intensive care unit

### Rates of severe or critical cases

**Fig. 3:**
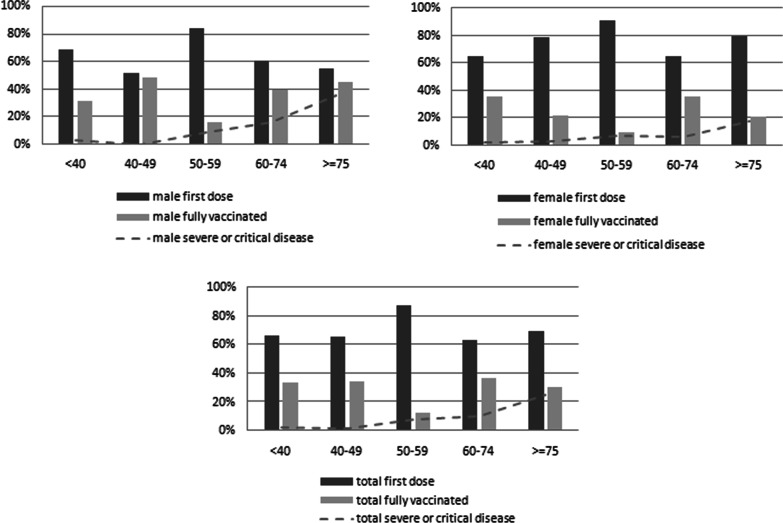
Distribution of severe or critical disease of COVID-19 by age, gender and vaccination status

Vaccine Breakthrough infections were considered severe or critical cases if the disease required hospitalization or led to death. The prevalence of patients under oxygen was 6.9%, whereas 2.1% were admitted toICUs, and 1.8% of COVID-19 cases led to death. The prevalence of severe or critical cases in vaccinated COVID-19 patients occurs in 10.8% of cases. The proportion of severe or critical cases varied by age groups with the highest for the group aged ≥ 75 years (24.5%) and the lowest for the group aged 40–49 years. We also observed higher proportions of severe or critical cases in males aged ≥ 75 years and 60–74 years (35% and 16% respectively) than females in the same age groups (17% and 6% respectively). In addition, proportions of the first dose were higher in females aging 60–74 years and ≥ 75 years (65% vs 79% respectively) than males (61% vs 55% respectively) whereas the proportion of fully vaccinated males in the same age groups (39% and 45% respectively) were higher than females (35% vs 21% respectively). Figure 3 shows the distribution of severe or critical disease of COVID-19 by age, gender and vaccination status.

### Factors associated with severe or criticalCOVID-19 cases among the vaccinated population

Table 3 shows the unadjusted and adjusted odds of severe or critical cases of COVID-19. In the adjusted analysis (model 2), the male gender was associated with approximately twice the odds of severe or critical cases as the female gender (odds ratio, 2.14; 95% confidence interval [CI], 1.15 to 3.9). In addition, increasing age, having a medical history of cardiovascular diseases, and being vaccinated with one dose of vaccine, were associated with an increased odd of severe or critical cases, whereas a higher score of self-estimation of adherence to preventive was associated with lower odds of severe or critical cases.

**Table 3:** Unadjusted and Adjusted odds ratios for severe or critical disease of covid-19 in those vaccinated

	Severe or critical disease	Asymptomatic or mild disease	Model 1 OR(IC95%)	p-value	Model 2 OR(IC95%)	p-value
Total	56 (10.8)	562 (89.2)	–	–	–	-
Age	65.2 (13.0)	55.0 (14.0)	1.06 (1.03–1.08)	< 10^–3^	1.05 (1.02–1.07)	0.001
Gender						
Female	23(06.0)	327 (94.0)	Reference	0.024	Reference	
Male	33 (11.1)	237 (88.9)	1.95 (1.09–3.49)		2.14 [1.15–3.90]	0.015
Employment						
Healthcare worker	8 (06.6)	114 (93.4)	Reference	0.458	–	-
Others	43 (08.6)	456 (91.4)	0.74 (0.34–1.62)		–	-
Medical history						
Absence	13 (04.1)	304 (95.9)	Reference		Reference	
CVD	26 (15.0)	147 (85.0)	4.13 (2.06–8.28)	< 10^–3^	2.70 [1.21–5.99]	0.015
Others*	12 (09.2)	119 (90.8)	2.35 (1.04–5.31)	0.039	1.98 [0.82–4.72]	0.124
Tobacco use						
Smokers	10 (09.0)	101 (91.0)	Reference	0.736	–	-
Non-smokers	41 (08.0)	469 (92.0)	0.88 (0.42–1.82)		–	-
Self-estimation of adherence to preventive measure						
Poor (score ≤ 4)	11 (19.0)	47 (81.0)	Reference		Reference	
Medium (5 ≤ score ≤ 7)	24 (07.4)	299 (92.6)	0.34 (0.15–0.74)	0.007	0.28 [0.11–0.73]	0.001
High (score ≥ 8)	16 (06.7)	224 (93.3)	0.30 (0.13–0.70)	0.005	0.29 [0.12–0.69]	
Vaccination schema						
Complete	10 (05.8)	162 (94.2)	Reference		Reference	0.007
Uncomplete	41 (09.1)	408 (90.9)	1.62 (0.97–3.32)	0.181	3.01 (1.34–6.78)	
Vaccine						
Astrazeneca	9 (15.8)	48 (84.2)	Reference	0.031	–	–
Others	42 (07.5)	516 (92.5)	2.3 (1.05–5.01)		–	–

*Model 1* unadjusted model, *Model 2* adjusted model

## Discussion

To the best of our knowledge, this study is the first to report the above associations in a cohort of vaccinated subjects in Tunisia. In addition, this is a census survey, COVID19 incidence calculated in this study is representative of all the population of the governorate of Sousse. Our study showed that the COVID-19 incidence was higher among people receiving an incomplete vaccination schema compared to fully vaccinated persons consistent with findings in other studies. According to the vaccine type, the highest incidence rate was observed among persons having been vaccinated with the Sputnik V vaccine, and the least incidence was observed with the AstraZeneca vaccine. This finding highlights the discrepancy in the effectiveness of each vaccine on the prevention of transmission potential in the wider populations with different local characteristics and virus variants of COVID-19 infection [21].

Stouten and colleagues [22] found that adenoviral-vector-based vaccines were associated with a higher risk of breakthrough infections, compared to mRNA-based vaccines. In a large real-world cohort of patients on dialysis, Brunelli and colleagues [23] highlighted an inconsistent antibody response in Ad26.COV2.S comparing with BNT162b2, however, no difference was detected in clinical effectiveness over generali the first 6 months postvaccination. However, these findings should be interpreted with caution since the different vaccine types were introduced in different times through the COVID-19 epidemic.

Almost 72.3% of the study population received one dose whereas only 27.7% were fully vaccinated. In addition, the prevalence of severe or critical cases in vaccinated COVID-19 patients occurs in 10.8% of cases, which is in accordance with some of the few studies in the literature [7, 13, 24]. However, The finding for our cohort (10.8%) was much higher than the prevalence (0.5%) observed in previous research between 15 December 2020 and 30 June 2021 in US Veterans Health Administration (VA) healthcare system [25]. This inconsistency may be due to the fact that several factors are interrelated in the occurring of severe COVID-19 disease after vaccination [26]. Yet, these factors could differ and thus influence the rate of serious COVID-19 breakthrough infection from one region to another.

Our current study presents multiple valuable COVID-19-related associations in the group of vaccinated COVID-19 patients during the study period. First, critical COVID-19 disease was noticed in older age groups regardless of vaccination status. Second, the severe or critical cases rate of 35% and 16% was significantly higher among males in the age classes 60–74 years and ≥ 75 years respectively than female participants (17% and 6% respectively) although the percentage of fully vaccinated patients was higher in males than females in these categories of age. Findings in the literature indicated that the male gender is a risk factor for serious COVID-19 disease that is explained by the differences in immunity response, the role of sex hormones, and gender-related behavior [25–27].

After adjustments, patients with a medical history of cardiovascular diseases had more than two times increased odds to have a serious disease. Immunocompromising conditions (such as hepatological disease, chronic inflammatory diseases, solid or hematological cancers, etc.) could weaken the antibody response to the vaccination [28]. Additionally, Wang et al. noticed between August 4 and October 12, 2021, a higher prevalence of severe breakthrough SARS-CoV-2 delta (B.1.617.2) variant era among patients with cardiovascular and lung disease, type 2 diabetes, history of malignancy, and baseline use of immunosuppressive medications [29].

Patients with cardiovascular diseases were more likely to have a serious disease. On the one hand, the effect of an unsettled immunity system on CVDs' exacerbation was demonstrated in the literature [30, 31]. On the other hand, dysregulated inflammatory response to COVID-19, more precisely neutrophil extracellular traps (NET) formations, induce immune-thrombosis and exacerbate inflammation [32]. These findings may explain the higher risk of developing serious cases of COVID-19 among individuals with CVD compared to individuals without CVD.

We also found the highest self-estimation of adherence to preventive measures was inversely correlated to severe or critical cases. Despite the efficacy of vaccination, preventive measures including the use of face masks, washing, or rubbing hands, and social distancing are crucial to reducing the virus spread whether in terms of speed or the transmitted viral load [24]. Lastly, having an incomplete vaccination schema was strongly associated with severe or critical breakthrough infection. Previous population-based studies showed that unvaccinated adults are far more likely to be hospitalized compared with fully vaccinated adults at least 14 days before the onset of the symptoms. in all adult age groups [7, 33, 34]. However, vaccination was less effective against hospital admission or death among old people compared to younger people [13], especially among those not fully vaccinated [8, 35]. Butt et al. found that the risk of severe outcomes of post-vaccination disease was higher with increasing age, having more than 4 comorbidities, while being vaccinated was associated with strong protection against severe or critical disease [25]. Besides, a previous study revealed that clinical symptoms of the SARS-CoV-2 infection among partially or fully vaccinated cases was significantly lower (odds ratio = 0.26 [95% CI: 0.07–0.94]) than in unvaccinated cases [36].

Dysregulation of immune function like inflammaging and immunosenescence can increase the vulnerability of subjects to COVID-19 and result in inefficient protection after vaccination [37].

Moreover, researchers suggested that the post-vaccination infections might be increasingly registered with the emerging variants of concern (VOCs) [38], which turn out to be less susceptible to neutralizing antibodies [39]. Nevertheless, a previous study suggested that breakthrough infection itself could substantially boost the vaccine-induced antibodies, especially the variant cross-neutralization [40]. There is limited research discussing the efficacy of antibodies cocktails [39], particularly with the high transmissibility of the Delta and Omicron variants [41].

Stopping the spread at the source mitigates opportunities for the virus to mutate by reducing the amount of viral transmission and therefore reducing the circulation of new variants [42]. With respect to the observation that most COVID-19 infections occurred nearly 4 months after launching the vaccination campaign, it was important to notice that during the fourth wave of COVID-19 infections the Delta variant was the main epidemic strain in Tunisia likewise globally [43]. A higher COVID-19 incidence rate was seen during the delta period than during the alpha period. Therefore, our findings may be not only attributable to vaccine effectiveness, but also to the variants of SARS-CoV-2 virus, different social restriction measurements and seasonal effects. Although the vaccination cannot prevent Delta variant infection and morbidity, worldwide data have revealed that the full course vaccinated population has been effectively protected against severe illness and death [44]. Moreover, a retrospective cohort study over 8 months by Tartof and colleagues [45] found that the effectiveness of BNT162b2 vaccine against COVID-19 decreased over time; yet, its effectiveness against hospital admission remained robust among fully vaccinated individuals.

Strengths of our study include evaluation of a regional sample with frequent testing of the population in the governorate of Sousse, availability of all vaccination and testing data in the comprehensive regional COVID- 19 confirmed cases database, subject interviews to confirm socio-demographic and clinical variables. Furthermore, the relatively considerable number of outcomes of interest and characteristics of the population which may be generalizable to other countries and regions.

Though we tried to provide evidence about the post-vaccination infections to improve measures of prevention and control of COVID-19, this study has some limitations.

As reported previously, the laboratory results may show false negatives and fortunately, this is not a frequent technical issue [46]. For testing all samples, several accredited public and private laboratories were authorized which may induce differences in the reliability of tests. Additionally, recall bias could alter the exactness of some information reported by cases. However, the trained investigators have spent sufficient time with each participant to enable them to take back memories and therefore minimize recall bias. Another limitation is that the vaccine may mitigate the symptoms of the SARS-CoV-2 infection. Herein, some asymptomatic people would escape from the COVID-19 screening. Moreover, we included only individuals over 19 years of age in the present analysis because they corresponded to the population initially targeted by the Tunisian vaccination campaign. Vaccination of children and adolescents was made possible at a much later stage (September 2021). Besides, the relatively short follow-up since the vaccination onset may affect the precisions of our estimates and our ability to evaluate the outcomes of breakthrough infections during the epidemic strain of Omicron VOC in the fifth wave. Hence, the generalizability of our findings needs to be validated in other populations including younger ages and other national data resources, and extended to the fifth wave exploring the Omicron VOC with assessing the potential waning after full vaccination. Evaluating serious outcomes of breakthrough infections beyond 8 months will be crucial for updating COVID-19 vaccine policy.

Additionally, comparing the severe outcomes with unvaccinated individuals warrant further investigation. Finally, additional studies should be conducted to quantify the real effectiveness of the combination of various control measures in the Tunisian population data and to provide further guidance on effectiveness differences according to variants of the SARS-CoV-2 virus and the booster shot indication to reduce the severe disease of breakthrough infections.

## Conclusions

Our study revealed a relatively low prevalence of severe or critical disease after breakthrough infections among the population of the governorate of Sousse. Furthermore, we investigated the correlation of age, gender, being a healthcare worker, medical history, tobacco use, self-estimation of adherence to preventive measure, vaccination status, and vaccine type with the severity of the COVID-19 disease. Our findings suggested that having cardiovascular disease, poor self-estimation of adherence to preventive measures, and incomplete vaccination schema were important to predict the severity of SARS-CoV-2 infection among a vaccinated Tunisian population. Therefore, boosting immunity for vulnerable patients added to maintaining and promoting preventive measures are not only essential to prevent severe cases of breakthrough infections of COVID-19, but also other influenza-like diseases.

## Data Availability

The datasets used and/or analysed during the current study are available from the corresponding author on reasonable request. The datasets generated and/or analyzed during the current study are not publicly available due to privacy of data but are available from the corresponding author on reasonable request.

## Abbreviations

COVID-19: Coronavirus disease 2019
SARS-CoV-2: Severe acute respiratory syndrome coronavirus 2
RT-PCR: Reverse Transcriptase Polymerase Chain Reaction
SIR: Specific incidence rate
ICU: Intensive care unit
95% CI: 95%Confidence interval

## References

[1] World Health Organization. Coronavirus (COVID-19) Dashboard. https://covid19.who.int. Accessed 4 Dec 2021

[2] Torjesen I (2021). Covid-19 will become endemic but with decreased potency over time, scientists believe. BMJ.

[3] Hasan T, Beardsley J, Marais BJ, Nguyen TA, Fox GJ (2021). The implementation of mass-vaccination against SARS-CoV-2: a systematic review of existing strategies and guidelines. Vaccines.

[4] Ritchie H, Mathieu E, Rodés-Guirao L, Appel C, Giattino C, Ortiz-Ospina E, et al. Coronavirus Pandemic (COVID-19). Our World Data. 2020. https://ourworldindata.org/covid-vaccinations. Assessed 6 Dec 2021.

[5] What are whole virus vaccines and how could they be used against COVID-19. https://www.gavi.org/vaccineswork/what-are-whole-virus-vaccines-and-how-could-they-be-used-against-covid-19. Assessed 5 Dec 2021.

[6] Paltiel AD, Schwartz JL, Zheng A, Walensky RP (2021). Clinical outcomes of A COVID-19 vaccine: implementation over efficacy. Health Affairs (Project Hope).

[7] Haas EJ, Angulo FJ, McLaughlin JM, Anis E, Singer SR, Khan F (2021). Impact and effectiveness of mRNA BNT162b2 vaccine against SARS-CoV-2 infections and COVID-19 cases, hospitalizations, and deaths following a nationwide vaccination campaign in Israel: an observational study using national surveillance data. Lancet.

[8] Lopez Bernal J, Andrews N, Gower C, Robertson C, Stowe J, Tessier E (2021). Effectiveness of the Pfizer-BioNTech and Oxford-AstraZeneca vaccines on covid-19 related symptoms, hospital admissions, and mortality in older adults in England: a test-negative case-control study. The BMJ.

[9] Chodick G, Tene L, Patalon T, Gazit S, Ben Tov A, Cohen D (2021). Assessment of effectiveness of 1 dose of BNT162b2 vaccine for SARS-CoV-2 infection 13 to 24 days after immunization. JAMA Netw Open.

[10] Christie A, Henley SJ, Mattocks L, Fernando R, Lansky A, Ahmad FB (2021). Decreases in COVID-19 cases, emergency department visits, hospital admissions, and deaths among older adults following the introduction of COVID-19 vaccine—United States, September 6, 2020–May 1, 2021. MMWR Morb Mortal Wkly Rep.

[11] White EM, Yang X, Blackman C, Feifer RA, Gravenstein S, Mor V (2021). Incident SARS-CoV-2 infection among mRNA-vaccinated and unvaccinated nursing home residents. New Engl J Med..

[12] Graham MS, Sudre CH, May A, Antonelli M, Murray B, Varsavsky T (2021). Changes in symptomatology, reinfection, and transmissibility associated with the SARS-CoV-2 variant B117: an ecological study. Lancet Public Health..

[13] Scobie HM, Johnson AG, Suthar AB, Severson R, Alden NB, Balter S (2021). Monitoring incidence of COVID-19 cases, hospitalizations, and deaths, by vaccination status—13 US jurisdictions, April 4–July 17, 2021. MMWR.

[14] Griffin JB, Haddix M, Danza P, Fisher R, Koo TH, Traub E, Gounder P, Jarashow C, Balter S (2021). SARS-CoV-2 infections and hospitalizations among persons aged ≥16 years, by vaccination status—Los Angeles County, California, May 1–July 25, 2021. MMWR.

[15] Havers FP, Pham H, Taylor CA, Whitaker M, Patel K, Anglin O (2021). COVID-19-associated hospitalizations among vaccinated and unvaccinated adults ≥18 years—COVID-NET, 13 states, January 1–July 24, 2021. MedRxiv.

[16] Ministry of Health. Coronavirus. Tunis, 2021. http://coronavirus.rns.tn/. Assessed 8 Dec 2021.

[17] Ministry of Health. Vaccination Strategy against covid-19. Tunis, 2021. http://www.santetunisie.rns.tn/images/strategie-vaccination-covid-19.pdf. Assessed 13 Nov 2021.

[18] Ministry of Health. National Dashboard of coronavirus Vaccination. Tunis, 2021. https://evax.tn/vaccinationOD.html. Assessed 13 Nov 2021.

[19] National Institute of Statistics. Statistics of Tunisian population. Tunis, 2021. http://www.ins.tn/statistiques/111. Assessed 12 Oct 2021.

[20] National Observatory of New and Emerging Diseases. Definition of COVID-19 cases in Tunisia. Tunis, 2020. https://www.onmne.tn/?p=10305. Assessed 20 Jan 2022.

[21] Olliaro P, Torreele E, Vaillant M (2021). COVID-19 vaccine efficacy and effectiveness—the elephant (not) in the room. Lancet Microbe.

[22] Stouten V, Hubin P, Haarhuis F, van Loenhout JAF, Billuart M, Brondeel R (2022). Incidence and risk factors of COVID-19 vaccine breakthrough infections: a prospective cohort study in Belgium. Viruses.

[23] Brunelli SM, Sibbel S, Karpinski S, Marlowe G, Walker AG, Giullian J (2022). Comparative effectiveness of mRNA-based BNT162b2 vaccine versus adenovirus vector-based Ad26COV2S vaccine for the prevention of COVID-19 among dialysis patients. J Am Soc Nephrol.

[24] Bosch W, Cowart JB, Bhakta S, Carter RE, Wadei HM, Shah SZ (2021). COVID-19 vaccine-breakthrough infections requiring hospitalization in mayo clinic florida through August 2021. Clin Infec Dis..

[25] Butt AA, Yan P, Shaikh OS, Mayr FB, Omer SB (2021). Rate and risk factors for severe/critical disease among fully vaccinated persons with breakthrough severe acute respiratory syndrome coronavirus 2 (SARS-CoV-2) infection in a high-risk national population. Clin Infec Dis..

[26] Lipsitch M, Krammer F, Regev-Yochay G, Lustig Y, Balicer RD (2022). SARS-CoV-2 breakthrough infections in vaccinated individuals: measurement, causes, and impact. Nat Rev Immunol.

[27] Gebhard C, Regitz-Zagrosek V, Neuhauser HK, Morgan R, Klein SL (2020). Impact of sex and gender on COVID-19 outcomes in Europe. Biol Sex Differ.

[28] Lakbar I, Luque-Paz D, Mege J-L, Einav S, Leone M (2020). COVID-19 gender susceptibility and outcomes: a systematic review. PLoSONE.

[29] Scully EP, Haverfield J, Ursin RL, Tannenbaum C, Klein SL (2020). Considering how biological sex impacts immune responses and COVID-19 outcomes. Nat Rev Immunol.

[30] Agca R, Smulders Y, Nurmohamed M (2022). Cardiovascular disease risk in immune-mediated inflammatory diseases: recommendations for clinical practice. Heart.

[31] Jaén RI, Val-Blasco A, Prieto P, Gil-Fernández M, Smani T, López-Sendón JL (2020). Innate immune receptors, key actors in cardiovascular diseases. JACC Basic Transl Sci.

[32] Li Q, Wang Y, Sun Q, Knopf J, Herrmann M, Lin L (2022). Immune response in COVID-19: what is next?. Cell Death Differ.

[33] Kearns P, Siebert S, Willicombe M, Gaskell C, Kirkham A, Pirrie S, et al. Examining the immunological effects of COVID-19 vaccination in patients with conditions potentially leading to diminished immune response capacity—the OCTAVE Trial. Rochester, NY: Social Science Research Network; 2021. Report No.: ID 3910058.

[34] Wang SY, Juthani PV, Borges KA, Shallow MK, Gupta A, Price C (2022). Severe breakthrough COVID-19 cases in the SARS-CoV-2 delta (B16172) variant era. Lancet Microbe..

[35] Tran TNA, Wikle NB, Albert E, Inam H, Strong E, Brinda K (2021). Optimal SARS-CoV-2 vaccine allocation using real-time attack-rate estimates in Rhode Island and Massachusetts. BMC Med.

[36] Bhattacharya A, Ranjan P, Ghosh T, Agarwal H, Seth S, Maher GT (2021). Evaluation of the dose-effect association between the number of doses and duration since the last dose of COVID-19 vaccine, and its efficacy in preventing the disease and reducing disease severity: a single centre, cross-sectional analytical study from India. Diabetes Metab Syndr.

[37] Alcendor DJ, Matthews-Juarez P, Smoot D, Hildreth JE, Lamar K (2022). Breakthrough COVID-19 infections in the US: implications for prolonging the pandemic. Vaccines.

[38] Chung H, He S, Nasreen S, Sundaram ME, Buchan SA, Wilson SE (2021). Effectiveness of BNT162b2 and mRNA-1273 covid-19 vaccines against symptomatic SARS-CoV-2 infection and severe covid-19 outcomes in Ontario, Canada: test negative design study. The BMJ.

[39] Li L, Han Z-G, Qin P-Z, Liu W-H, Yang Z, Chen Z-Q (2022). Transmission and containment of the SARS-CoV-2 delta variant of concern in Guangzhou, China: a population-based study. PLoS Negl Trop Dis.

[40] Bernal JL, Andrews N, Gower C, Gallagher E, Simmons R, Thelwall S (2021). Effectiveness of Covid-19 vaccines against the B.1.617.2 (Delta) variant. New Engl J Med.

[41] Chen RE, Zhang X, Case JB, Winkler ES, Liu Y, VanBlargan LA (2021). Resistance of SARS-CoV-2 variants to neutralization by monoclonal and serum-derived polyclonal antibodies. Nat Med.

[42] World Health Organization. The effects of virus variants on COVID-19 vaccines. Geneva, 2021. https://www.who.int/news-room/feature-stories/detail/the-effects-of-virus-variants-on-covid-19-vaccines. Accessed 30 Aug 2022

[43] Chouikha A, Fares W, Laamari A, Haddad-Boubaker S, Belaiba Z, Ghedira K (2022). Molecular epidemiology of SARS-CoV-2 in Tunisia (North Africa) through several successive waves of COVID-19. Viruses.

[44] Bian L, Gao Q, Gao F, Wang Q, He Q, Wu X (2021). Impact of the Delta variant on vaccine efficacy and response strategies. Expert Rev Vaccines.

[45] Tartof SY, Slezak JM, Fischer H, Hong V, Ackerson BK, Ranasinghe ON (2021). Effectiveness of mRNA BNT162b2 COVID-19 vaccine up to 6 months in a large integrated health system in the USA: a retrospective cohort study. Lancet Lond Engl.

[46] Bates TA, McBride SK, Winders B, Schoen D, Trautmann L, Curlin ME (2022). Antibody response and variant cross-neutralization after SARS-CoV-2 breakthrough infection. JAMA.

